# Supplementary material for: A Radiomics Approach on Chest CT Distinguishes Primary Lung Cancer from Solitary Lung Metastasis in Colorectal Cancer Patients
Source: J Pers Med. 2022 Nov 7;12(11):1859. doi: 10.3390/jpm12111859 (PMC9695650; doi:10.3390/jpm12111859)
Supplement: Supplementary file 1 [file jpm-12-01859-s001.zip › jpm-1949044-supplementary.pdf]

Supplementary Table S1. The list of the radiomic features that were selected for the SVM classifier and their ICC results.

Radiomic features	Feature type	Primary LC	Solitary LM	*P* value[†]	Inter-reader ICC[*]
**Intranodular radiomic feature**					
Sphericity	Shape	0.63 ± 0.08	0.73 ± 0.06	<0.001	0.852 (0.798, 0.892)
GLCM Correlation	Texture	0.26 ±0.16	0.09 ± 0.16	<0.001	0.874 (0.828, 0.908)
GLDM Dependence Entropy	Texture	6.56 ± 0.39	6.16 ± 0.38	<0.001	0.936 (0.912, 0.953)
**Perinodular radiomic feature**					
GLDM Dependence Non-Uniformity Normalized	Texture	0.23 ± 0.06	0.21 ± 0.04	0.009	0.899 (0.861, 0.926)

Note: Values are presented as mean ± standard deviation where applicable. SVM, support vector machine; ICC, intraclass correlation coefficient; GLCM, grey level co-occurrence matrix; GLDM, gray level dependence matrix.

[†]*P* values are for comparisons between primary LC and solitary LM.

[*]Values in parentheses are 95% confidence intervals.

Supplementary Table S2. Semantic CT imaging features of solitary pulmonary nodules in the training, internal, and external testing datas.

	**Training (*n*=159)**	**Internal testing (*n*=40)**	**External testing (*n*=40)**	***P* value**
**Size (mm)**	17.2 ± 7.0	17.0 ± 9.6	16.0 ± 7.0	0.628
**Margin**				0.259
Smooth	53 (33.3)	14 (35.0)	20 (50.0)	
Lobulated	72 (45.3)	20 (50.0)	12 (30.0)	
Spiculated	34 (21.4)	6 (15.0)	8 (20.0)	
**Density**				0.221
Solid	138 (87.4)	38 (95.0)	33 (82.5)	
Subsolid	20 (12.6)	2 (5.0)	7 (17.5)	
**Air-bronchogram**				**0.043***
Absent	126 (79.2)	38 (95.0)	35 (87.5)	
Present	33 (20.8)	2 (5.0)	5 (12.5)	
**Cavitation**				**<0.001****
Absent	146 (91.8)	28 (70.0)	33 (82.5)	
Present	13 (8.2)	12 (30.0)	7 (17.5)	
**Pleural tag**				0.631
Absent	106 (66.7)	29 (72.5)	25 (62.5)	
Present	53 (33.3)	11 (27.5)	15 (37.5)	

Note: Values in parentheses are percentages. Values are presented as mean ± standard deviation where applicable. Significant *P* values are in bold. SPN, solitary pulmonary nodule

*Post-hoc analysis was performed to compare the proportion of SPNs with air-bronchogram between the three datas, the proportion of SPNs with air-bronchogram was significantly higher in the training data than in the internal testing data.

**Post-hoc analysis was performed to compare the proportion of SPNs with cavitation between the three datas, the proportion of SPNs with cavitation was significantly higher in the internal testing data than in the training data.
